# The impact of libraries and informationists on patient and population care: a mixed-methods study

**DOI:** 10.5195/jmla.2024.1520

**Published:** 2024-01-16

**Authors:** Carol Shannon, Jacqueline L. Freeman, Mark MacEachern, Gurpreet K. Rana, Craig Smith, Judith E. Smith, Jean Song

**Affiliations:** 1 cshannon@umich.edu, Informationist, Taubman Health Sciences Library, University of Michigan, Ann Arbor, MI; 2 jlfreem@umich.edu, Informationist, Taubman Health Sciences Library, University of Michigan, Ann Arbor, MI; 3 maceachern.mark@gmail.com, Informationist, Taubman Health Sciences Library, University of Michigan, Ann Arbor, MI; 4 preet@umich.edu, Informationist, Taubman Health Sciences Library, University of Michigan, Ann Arbor, MI; 5 craigsm@umich.edu, Assessment Specialist, University Library, University of Michigan, Ann Arbor, MI; 6 judsmith@umich.edu, Informationist, Taubman Health Sciences Library, University of Michigan, Ann Arbor, MI; 7 jeansong@umich.edu, Director, Mardigian Library, University of Michigan - Dearborn, Dearborn, MI

**Keywords:** Assessment, population health, mixed methods, patient care, libraries, librarians

## Abstract

**Objective:**

While several studies have examined the effectiveness of librarian interactions with clinicians and impact of librarians on patient care, no studies have explored a library's effects on population care. The goal of this study was to investigate the library's impact on both patient and population care.

**Methods:**

Using a sequential exploratory mixed-methods design, we first interviewed a small set of clinicians and researchers active in patient and population care. Based on the themes that we discovered through coding the interviews, we created a survey that was sent to faculty in the health sciences and the health system.

**Results:**

We collected data from a representative sample of our population. We discovered that all respondents value the library and informationists, using our services most for teaching, publishing, presenting, and professional development.

**Conclusion:**

We now have data to support our value to our population and to show where we can do more work to improve the use of our services. Our study shows the value of doing a mixed-methods sequential exploration in which themes that are important to our user community were identified prior to launching a large-scale survey.

## INTRODUCTION

At the Taubman Health Sciences Library (THL) at the University of Michigan, informationists work closely with researchers, faculty, staff, clinicians, and students who focus on the health of patients and populations in the schools of dentistry, medicine, nursing, pharmacy, and public health, as well as the health system (Michigan Medicine). We partner with our users in a variety of ways: collaborating with teams that create local practice guidelines for the health system; supporting health systems researchers; providing instruction in evidence-based practice; and participating in evidence syntheses research projects. However, because we are not clinical librarians who work on the wards, we rarely get the opportunity to directly observe the impact of our library on patient care. While we have long had anecdotal evidence that our community strongly values our expertise and knowledge, we sought to measure and understand the library's impact in both patient and population care, to make meaningful decisions on resources both human and financial.

The THL Assessment Working Group was convened to conduct the project. We began with a review of the

We used the following definition of population care in our study and provided it to all who completed either an interview or the survey.

Population care can be defined as health care for broader populations rather than individuals and can include concepts such as clinical guidelines, protocols and legal policy. Specifically, for the purposes of this study, we define population care as “the health care needs of a specific population and making health care decisions for the population as a whole rather than for individual” [11].

In light of this information gap that we discovered through our literature review, we designed a mixed-methods study to understand our impact on both patient and population care. Our use of the word “impact” follows a common usage in the literature that, for example, investigates factors as variables in health or information literacy. Information is but one element in the long process of patient care or the implementation of policy and never directly touches the patient or the population, making it extremely difficult to measure.

## METHODS

### Phase 1

**Figure 1:**
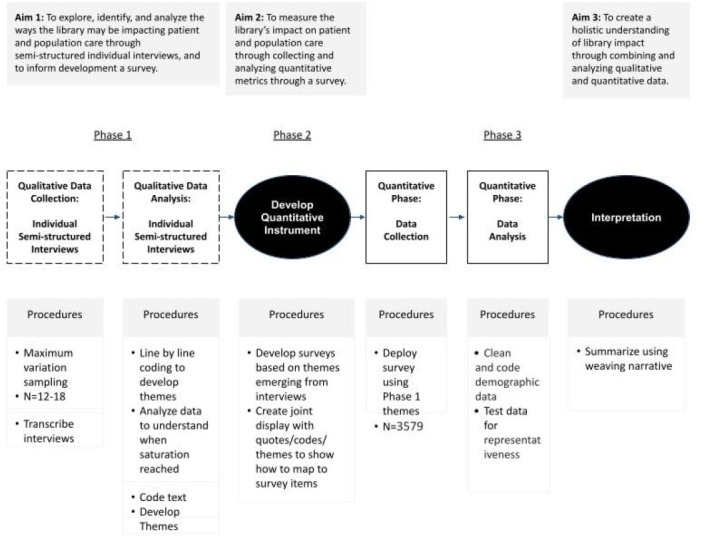
Mixed-methods sequential exploratory study design

We selected a mixed-methods approach for this project, so that we could utilize the strengths of both qualitative and quantitative data: the data we collected would create a holistic understanding of our impact on patient and population care in a way that could not be achieved by using one type of data alone. The exploratory sequential study design, which includes a qualitative phase followed by a quantitative phase that is informed by the qualitative phase, was the best fit for our study. Figure 1 summarizes our exploratory sequential design [12].

The University of Michigan Health Sciences and Behavioral Sciences IRB reviewed the project and determined that the “study does not fit the definition of human subjects research” and would not need to be regulated [HUM00131285].

The goal of Phase 1 was to use individual, semi-structured interviews to inform our survey instrument, which was based on Marshall [9], but also would be influenced by our two pilot in-person interviews. In these interviews we had two objectives: 1) to broadly explore themes so that we could better understand ways in which the library may be impacting patient and population care and 2) to inform the creation of the quantitative survey. We created an interviewer handbook, protocol, and list of codes that informed the development of our tools, also modeled on Marshall [9]. The codes that we used were divided into categories such as Roles, Resources, and Informationist Integration, which were further defined by subheadings, including Point of care, Research, and Authorship.

The questions for the qualitative portion of the study included asking interviewees to describe their work (either patient or population care or both), their own use of resources, their experiences of working with an informationist, and how the informationist contributed to their work.

The target population consisted of University of Michigan health care providers and researchers across the health sciences who use the library and either provide patient care or conduct work that explicitly attempts to have an impact on population care, such as health policy or clinical guidelines. Students (including resident physicians) were excluded from the study because they do not have final responsibility for patient care.

To identify potential stakeholders to interview, we conducted a survey of THL informationists to collect names of faculty members to participate in these initial interviews. The recommending informationist then reached out to the faculty member to ensure a better uptake of the interview invitations.

We drafted the Phase 1 questionnaire using probes from Creswell [13], such as “Tell me more;” “What is an example of that?;” and “Could you explain your response more?” We piloted the questionnaire during interviews with two faculty members. We then revised it for use in the 11 formal interviews. The questionnaire is available in the material found under the Data Availability Statement. We included definitions for “population care,” “informationist,” and “institution,” terms that we felt might not be familiar to or defined in the same way by everyone.

Two team members individually conducted the interviews, which were scheduled for 40 minutes. The pilot interviews were used only to test the survey instrument, and we did not retain the data from them for analysis. All interviews were recorded and transcribed using Scribie [14]. They were coded independently by two of the report authors. Coding, using a combination of codes from Marshall [9] and codes that emerged from the interviews, was conducted at the phrase and sentence level. The team members then compared their coding to resolve differences in how the codes were understood and applied and to address the emergence of new codes and incorporate them both retroactively and prospectively. Coding was done manually (on paper, using highlighting) by one team member and in Dedoose [15] by the other team member. Dedoose was also used to perform quantitative analyses.

### Phase 2

The COVID-19 pandemic had an extraordinary impact on health and healthcare, as well as the general population. It began while the Phase 2 questionnaire was being developed and approximately six months before that questionnaire was to be released. Because we wondered how this crisis might affect our study population, we added new questions to the survey to understand what effects, if any, the COVID-19 pandemic had on library resource use and access, and on patient and population care.

#### Gathering the Quantitative Data

Survey invitations were sent to 3,579 people identified through the University's data warehouse as having an affiliation with the schools/colleges of dentistry, medicine, nursing, pharmacy, public health, the hospital system, and also have a faculty appointment. The survey contained multiple-choice, Likert-scale, and open-response questions. It was administered online, using QualtricsXM software [16], over a three-week period in August 2020. Data were queried using IBM's SPSS Statistics [17].

We included demographic questions to understand who THL serves and to discover any gaps in how services were provided at the various schools, at the department level, at specific locations, by gender, and by race or ethnicity. We cleaned and coded the demographic data so that the sample could be tested for representativeness. We asked survey participants about their use of library services and collections in three theme areas, as well as library support during the COVID-19 pandemic.

## RESULTS

A strength of mixed-methods methodology is the intentional integration of the qualitative and quantitative data to create a holistic view of the data. Three overarching themes, with subthemes that we investigated in some detail, emerged from the qualitative data and were further explored in the quantitative survey.

1. Access to information resources (which can be further delineated as resource access, resource types, and resource use).
  “I feel that everything is literally at my fingertips. I can't tell you the last time I tried to pull an article from a journal that I couldn't access. So I think that that speaks to the depth and breadth of what the library has to offer, 'cause in my kind of work life, the things that I may look into are… Could be very diverse, and I've never had an issue getting an article, old, new, in an obscure journal versus a very mainstream journal. So I think that from a patient care perspective, if I'm about to do a procedure or I know I have a procedure coming up in the coming days and I wanna look up some stuff on it, it's very easy” (Participant #8, patient care provider).
2. Informationist integration and value (which can be itemized as awareness and connection, instruction, expert searching, and statements of informationist value).
  “Because this is a relatively new mandate, we've done a lot of benchmarking, a lot of lit reviews of how hospitals have approached this or how other institutions, Public Health, other groups, have approached this work. So library resources have been critical in just understanding what has been done to date in order to guide our feet in determining the best strategy for us. So just to get a lay of the land, to understand what the most fruitful direction to take should be, being at such a resource-rich place like U of M and having the help and support of informationists as well, the Public Health core has been just wonderful” (Participant #1, population care researcher).
  “And so whereas, like I said, there's not a specific patient encounter where I've called an informationist, I think that they've clearly shaped the care I deliver as a result of the education I've gotten over the last number of years being a part of this institution” (Participant #7, patient care provider).
3. Information seeking behaviors.
  “But I was at another institution for a few years before this that did not have this kind of support. And it really makes a difference in terms of your ability to stay up to date with things” (Participant #3, population care researcher).

The themes from Phase 1 informed the creation of questions for the Phase 2 survey and each theme is mapped to at least one survey question. Table 1 provides a selection of the themes from this phase, illustrative quotes from the interviews, and mapping to survey questions and results in Phase 2.

**Table 1:** Joint display of themes identified in Phase 1 qualitative interviews, examined in Phase 2 quantitative survey

Phase 1 Themes	Interview Quotations	Phase 2 Questionnaire
Access to information resources	“For guidelines, I'm tracking down guideline articles…for educational instruction, I'm often going back looking for articles in Academic Medicine and other places that have been cited to get the fuller reference and access to them.” (Participant #4, patient care provider)	Please select up to three types of information resources that are most important to your professional work. Journals (n=368), Evidencebased information resources (n=225), and Guidelines (n=197) were the top choices.
Informationist integration and value	“We have a bunch of clinicians trying to write grants when most of what they do is see patients, it doesn't come out so well without the help of the informationist I would say.” (Participant #9, patient care provider) “ [W]ithout the informationist we don't have the good background and we can't support our argument that a service is needed. If we can't support the argument that the service is needed then the service isn't there, and then the patients don't benefit from it…] (Participant #9, patient care provider)	How would you characterize the contribution of the informationist(s) to your information-seeking efforts? Of those who had an interaction with an informationist, 98% (n=128) found the interaction to be “very beneficial” or “somewhat beneficial”
Information Seeking Behavior	“…based on my role…I stay abreast with current evidence that's coming out in the scholarly journals to make sure that I'm treating my patients with the most up to date care and recommendations. Making the recommendations based off of evidence and not just based off of my experience, for example.” (Participant #3, population care researcher)	How do the resources you selected [as the most important to your professional work] impact your work? Teaching (n=345) Publishing and presenting (n=339) Professional development (n=321)

In Phase 1, the three roles (clinical, population, or clinical and population) were roughly equally represented among the interviewees (four, four, and three, respectively); however, how the participants answered questions varied significantly in relation to their role. We theorized that, with the expansion in the number of participants in Phase 2, we would see an even greater variation in the ways that respondents from each role used library resources and in the library's impact on patient and population care. We also were interested in finding any significant gaps in the provision of service or instances of high impact based on affiliations, locations, race or ethnic identity, and gender identity.

Due to attrition or bad contact information, 13 invitations failed to reach their intended recipient, leaving 3,566 active invitations. At the close of the survey, there were 506 completed questionnaires, for a response rate of 14.2%. Of the completions, 385 people selected a category describing their work as patient care (n=220), population care (n=54), or both patient and population care (n=111). We also collected data from participants who answered neither (n=121), as it provided useful information on the library's service provision; however, it is not the focus of this current study.

We tested data from the 385 eligible participants for representation against the population to which the survey was sent for school/college affiliation, gender, race or ethnicity, and appointment track (clinical, research, tenure, lecturer). In all areas except for gender there was a p-value of less than 0.05, indicating that there was no statistically significant difference between the eligible participants and the population invited to participate.

### Access to Information Resources

Phase 1 interviews provided an understanding of the range of library resources that clinicians and population care researchers find important to their work. Using their responses to populate a categorical list of resources and a free text “other” option, survey respondents in Phase 2 provided insights into which resources were used most often and how they were being used for patient care and population care research. In this study, respondents were asked to select up to three types of resources that were most important to their work. They selected journals (n=368), evidence-based resources (n=225), and guidelines (n=197) as their top choices. Reports (n=65) and statistical resources (n=65) ranked at the bottom of the list. Asked how the three chosen resources collectively impact their work, respondents indicated a range of activities whose relative ranks revealed a critical focus on instruction. Table 2 demonstrates how respondents most frequently used the top three resources ranked as important to their work.

**Table 2:** How do the information resources you selected impact your work?

Type of Impact	Frequency
Teaching	345
Publishing and presenting	339
Professional development	321
Decision making	316
Data work	227
Grants	208

### Informationist Integration and Value/Information Seeking

In Phase 1, we gathered rich qualitative data on how informationists impact the work of known library users. In Phase 2, we were able to quantify this impact among survey respondents who had a positive interaction with an informationist (categorized as “very beneficial” or “somewhat beneficial”) within the past three years (n=114). In terms of their impact, respondents noted that informationists improve efficiency, information seeking, and completion of work (Table 3).

**Table 3:** How do informationists help impact your work?

Type of Impact	Frequency	Percent
Improved my ability to find and use information	101	89
Contributed to the production and/or completion of my work	85	75
Helped me be more efficient with my time	73	64
Helped with my teaching and/or instruction needs	54	47
Informed my evidence-based decisionmaking process	31	27

### Use of Library Resources During COVID-19

Many libraries have been guided by their own understanding of their patrons' needs, by anecdotal evidence, and by usage statistics of the effects of the COVID-19 pandemic on their patrons' use of library resources [18, 19]. Survey respondents were asked if they were involved in work directly related to the pandemic, including online teaching, coronavirus research, or patient or population care (or both). Of those answering “yes” (n=274, 75%), 22% stated that their use of the library changed during the pandemic. We asked these respondents to briefly describe how their use of the library changed. Respondents wrote about the lack of access to the library's physical spaces: “Used to go to the physical library, but now no longer accessible—more dependent on internet resources” (Participant ID: R_2wF9UJQBvdZ5qme) and “Usually I use the great space for writing grants and papers, but that was not available during shutdown” (Participant ID: R_1CIohFnom8dZgyK).

Some decreases in the use of library resources due to the pandemic are beyond the library's control, such as: “[l]ess time to use library resources due to increase in clinical load,” (Participant ID: R_12PuETPGTEJYeXh) and “I would often interact online when I was in my office. During the pandemic I just didn't come to office [sic]” (Participant ID: R_8kddCh7AEFraQi5).

One respondent wrote about increased costs from being unable to access parts of the library collection: “Regret having limited access to textbooks; this required me to purchase them out of pocket” (Participant ID: R_1PTVRIoVysKpSSq).

The closure of physical spaces during the pandemic highlighted the ways that patrons, who use health sciences libraries and already interacting primarily with electronic collections before, continued to engage with library resources—some increasing their use—during the pandemic. The following quotes from the survey demonstrate the range of responses:

“[T]he online library resources were invaluable to not only patient care but also the ability to learn about and expand our understanding of COVID” (Participant ID: R_skjlOtQCAB0wNq1).

“More actively looking for peer reviewed data and clinical guidelines” (Participant ID: R_3ni421mjTKCfOfv).

“Increased use to write COVID-related grants” (Participant ID: R_1CBKg7YHn02Q2aE).

“Accessed more from home” (Participant ID: R_1fdOdJd1a7N13hr).

“Increased use of resources including library information lead to review and write articles around COVID-19 management” (Participant ID: R_1g6eKzuhPLqxOSc).

“I worked on a publication and accessed online library resources more during the pandemic” (Participant ID: R_1cZE1I6AcA7d5Q7).

## DISCUSSION

In the literature on surveys as a research methodology, females, including academic women, are more likely to respond to surveys [20] and, therefore, our respondent sample is like the population from which it was drawn across the major characteristics of this population. While the data demonstrated an appropriate representativeness, it should not be seen as characteristic of researchers and providers of patient and population care nationally. Rather, this data and the insights drawn from it provide a new understanding of the information behaviors and needs of this population and may be used to understand how libraries can approach engaging with their researchers and providers at similar large universities and affiliated healthcare systems.

The themes investigated in this study provide new insights into access to information resources, the integration and value of information professionals, and information-seeking behaviors that other health sciences libraries can use to support and deepen their engagement with researchers and providers of patient and population care. Additionally, the findings related to the use of library resources during the pandemic provide new information that health sciences librarians can use to understand how the use of resources was affected during COVID-19 and to plan for future extreme circumstances. At the moment, library literature describes how libraries addressed the challenges of continuing to provide services to their patrons providing patient care [21–23], the role of academic librarians in supporting the information needs of medical staff and researchers, and collaborations with physicians to provide critical and timely resources and intelligence reports [24, 25]. While this literature adds to what is known about how libraries responded to this emerging crisis, our study provides information about how providers of patient and population care used library resources during the pandemic and what they perceived as vital to their work.

Summative evaluations of informationists' work, such as when participants were asked about all the ways that informationist's help impacted their work, provide insight into areas of strongest impact. Using the SWOT analysis framework that considers areas of strength, weakness, opportunities, and threats, data from Phase 2 suggests that seeking additional opportunities to inform evidence-based decision-making through highlighting and providing instruction on the many evidence-based resources available through the library could enhance informationists' impact in this area. The data provide a guide to where future efforts to collaborate on patient and population care could see the largest increase in our engagement with this user group. While providers of patient and population care in this study did not perceive libraries and informationists as having as significant of an impact on informing their evidence-based decision-making processes, this could be due to 1) greater emphasis being placed on work we do to help them find and use information, 2) a lack of familiarity with our expertise in finding and synthesizing evidence, or 3) a researcher's previous negative experiences working with a librarian, whether at the University of Michigan or elsewhere.

This study offers insights into specific needs and concerns reported by providers of patient and population care during an emerging healthcare crisis that can be used to understand and plan for these needs ahead of the next interruption in regular service provision. Health sciences libraries can expect their patrons to be consumed by patient care responsibilities and the overall management of the population care crises while being simultaneously in need of library resources during the next emerging healthcare crisis. Creating tools that support efficient access to information, such as curated information portals guiding users to the most up-to-date research and implementing strategies for disseminating this information to patient and population care users during the next similar crisis are among the highest priorities information specialists can engage with. The closure of physical spaces to combat the spread of infectious disease reinforces the importance of access to online library resources, including access to informationist expertise. From instruction to research consultation to resource provision, each aspect of the library and informationists' work needs to function as seamlessly in the virtual environment as it does in person in order to best serve providers of patient and population care.

## LIMITATIONS

Respondents to the qualitative and quantitative components of the study may have been already favorably predisposed to the library, which may have resulted in disproportionately positive data. With the COVID-19 pandemic beginning about six months before the Phase 2 survey was released, there was probably an adverse effect on the number of survey responses that we received, since a willingness to take the survey would require time and energy on the part of providers and researchers, many of whom took on added burdens of research or patient care at that time. Thus, the study likely did not gather data from those most impacted by COVID-19 patient and population care responsibilities.

While the study sample was representative of the population from which it was drawn, it is not a representative sample of patient and population care providers and researchers nationally. Insights drawn from this sample may shed light on the information behaviors and needs of similar users at other large academic institutions with associated healthcare systems but are not meant to be generalizable.

## CONCLUSION

The impact of information resources on teaching highlighted in this study likely points to their importance at an academic institution where an emphasis on training health sciences professionals is part of the institution's mission. This has implications for the licensing of resources for both the academic and clinical environments and for considerations of cost and cost-sharing of resources between the library, health sciences schools, and clinical departments. Respondents in this study confirmed the importance of journal access for instruction, publishing and presenting, and professional development but seemed less aware of how working with informationists could inform their instruction or their evidence-based decision-making process. These latter two domains—with an emphasis on librarians' expertise in instruction on finding and using information, particularly for the evidence-based decision-making process—point to areas for increasing our impact on patient and population care.

Additionally, these data may be used to understand how to best support the work of patient and population care providers in a number of ways, including proactively looking for information competencies embedded within the health sciences schools' curricula and advocating for integration points for library instruction; working with departments to provide sessions on research dissemination, particularly in open access journals, which libraries have led the way in promoting; and sharing methods for creating alerts that help raise current awareness on topics key to ongoing professional development. Finally, using a mixed-methods research design allowed us to gather rich, multifaceted data. We had previous studies to look to for guidance, and some even used both qualitative and quantitative methods but not in a mixed-methods design. Using an explicit mixed-methods approach, in our case, the sequential exploratory mixed-methods design, meant that we could make informed decisions (based on the qualitative interviews) about the questions we would ask in our quantitative survey, rather than guessing or depending completely on the work of others. The mixed-methods approach is very useful for librarians, as it is flexible, providing multiple ways to gather both qualitative and quantitative data and a framework to integrate both types.

## Data Availability

Data associated with this article cannot be made publicly available because they contain personally identifiable information. Access to the data can be requested from the corresponding author and may be subject to IRB restrictions.

## SUPPLEMENTAL FILES

**Appendices:** The interview instrument and survey questionnaire are available at https://dx.doi.org/10.7302/7636.
